# Fungal and bat diversities along a landscape gradient in central Mexico

**DOI:** 10.1371/journal.pone.0310235

**Published:** 2024-09-09

**Authors:** Gabriel Gutiérrez-Granados, Uriel C. Torres-Beltrán, Judith Castellanos-Moguel, Ángel Rodríguez-Moreno, Víctor Sánchez-Cordero

**Affiliations:** 1 Facultad de Estudios Superiores Zaragoza, UMIEZ, UNAM, Batalla 5 de mayo s/n esquina Fuerte de Loreto, Col. Ejército de Oriente, CDMX, Iztapalapa, México; 2 Departamento El Hombre y Su Ambiente, Laboratorio de Micología, Universidad Autónoma Metropolitana-Xochimilco, CDMX, Mexico City, México; 3 Departamento de Zoología, Instituto de Biología, Pabellón Nacional de la Biodiversidad, Universidad Nacional Autónoma de México, Ciudad de México, Mexico City, México; University of Oklahama Norman Campus: The University of Oklahoma, UNITED STATES OF AMERICA

## Abstract

Species interactions between bats and fungi are poorly known. We documented the association between fungal and bat diversities along a landscape gradient. Ten, eight, and seven bat species were captured in conserved, semi-conserved, and urban sites, respectively. *Eptesicus fuscus*, *Myotis ciliolabrum* and *Corynorhinus townsendii* were the most abundant in conserved and semi-conserved sites. *E*. *fuscus*, *Myotis velifer*, and *Lasiurus cinereus* were abundant in urban sites. *C*. *townsendii* was the least abundant bat. A total of 15 cultivated fungi genera included the fungal diversity in bats, of which nine fungi genera were shared along the landscape gradient. *Penicillium* and *Aspergillus* were the most abundant genera, and *Aureobasidium*, *Bispora*, *Stachybotrys*, and *Verticillium* were only documented in the conserved sites. We observed a higher fungal diversity associated with bat species along this landscape gradient. The individual site-based accumulation curves of fungal diversity showed significant decreasing values along the conserved, semi-conserved, and urban sites, respectively. In conserved and urban sites, *M*. *californicus* and *M*. *velifer* showed the highest fungal diversity, respectively. *E*. *fuscus* was associated to the fungi genera *Scopulariopsis*, *Alternaria*, *Penicillium* and *Beauveria*; *L*. *cinereus* to *Cladosporium* and *Aspergillus*, and *M*. *velifer* to *Alternaria sp1*, *Bispora* and *Trichoderma*. Conserved sites showed both high bat and fungal diversities [species richness and abundance] compared to semi-conserved and urban sites. More studies associating bat and fungal diversities in other ecosystems are needed to corroborate this pattern.

## Introduction

Species interactions are central for understanding how animal communities interact with other biological groups, and interactions between faunistic and floristic groups have been extensively documented [1,2]. In particular, terrestrial mammals have been one of the faunistic groups where species interactions with different floristic groups have been widely studied [3], and this has been the case for bats [4,5]. However, species interactions between bats and fungi have been less studied, despite the fact that fungi comprise one the most diverse biological groups [6,7]. Studies relating species interactions between bats and fungi are restricted to a pathological perspective. For example, the association between the guano produced by bats, and the pathogenic fungus *Histoplasma capsulatum* affecting humans has received attention since almost 50 y ago [8–10]. More recently, species interactions between bats and the pathogen fungus *Pseudogymnoascus destructans* have raised concerns given the high mortality of bat populations in North America [11].

On the other hand, studies documenting species interactions between bats and fungi have been typically conducted in caves [8,11–13]. For example, there were 33 new fungi species documented in bat caves in China [14]. Nonetheless, it has also been documented that fungi species associated with bats live external to cave habitats, suggesting that both internal (caves) and external (outside caves) habitats contribute to bat potential mycobiota [15]. Further, bats increase the capacity of dispersion contributing to the presence of fungi in large geographical scales [16]. For example, the migratory bat *Tadarida brasiliensis mexicana* harbors a high fungal diversity in Colorado, including the genera *Aspergillus*, *Penicillium*, *Cladosporium* and *Eurotium* [17]. Holz [18] documented about 100 fungi genera on the bat *Miniopterus orianae* in Australia, where the most common fungus species was *Rhodotorula mucilaginosa*. Bats also host several airborne fungi, such as *Aspergillus* and *Penicillium*, which produce high quantities of spores and mycotoxins, resulting in allergies, mycosis, and mycotoxicosis in human populations [19]. These studies show the importance of bats and fungi species interactions involving high dispersal capabilities, and in some cases, fungi pathogens producing diseases in wildlife and humans [19].

Furthermore, it has been documented that habitat loss due to human-induced activities affect species diversities and interactions, respectively [20–22]. For example, species richness in bats decreases from conserved habitats to transformed habitats [23,24]. Several fungi groups are also susceptible to habitat loss and fragmentation, and loss of species richness of dispersal agents (as bats) results in a decrease of fungi species richness [25]. In this study, we anticipated that changes in bat diversity are correspondingly associated with changes in fungal diversity. Our aims were to document [1] an association between bat diversity and fungal diversity, and [2] changes of bat and fungal diversities associated along a landscape gradient, including conserved, semi-conserved, and urban habitats. We expected to observe a decrease in both bat and fungal diversity with decreasingly conserved landscapes.

## Methods

### Study site

**Fig 1:**
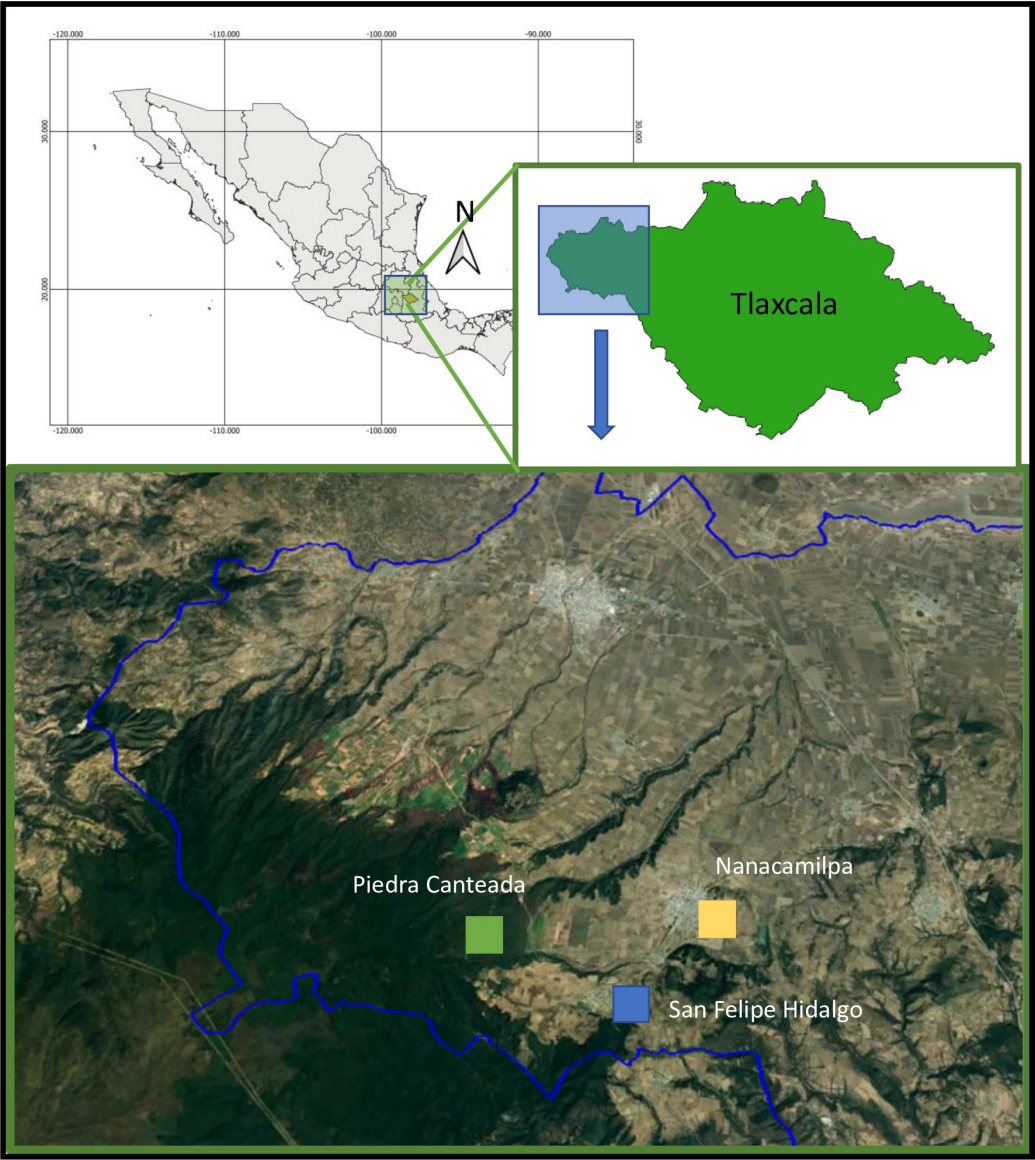
Map depicting the study site in central Mexico. Locality sites are shown in blue squares, along the landscape gradient. Piedra Canteada, conserved site; San Felipe Hidalgo, semi-conserved site, and Nanacamilpa, urban site. See Methods for details.

This study was conducted in the municipality of Nanacamilpa, State of Tlaxcala in central Mexico. Nanacamilpa has an altitudinal range of 2,300 to 3,400 m.a.s.l. with an average temperature of 18°C. Rainfall is from July to October, with an average annual precipitation of 80 mm (last checked April/2022; https://smn.conagua.gob.mx/es/climatologia/pronostico-climatico/precipitacion-form). Vegetation is dominated by pine forest (*Pinus teocote*). Three study sites were established along a landscape gradient including a [1] conserved pine forest site in Piedra Canteada, [2] semi-conserved pine forest site in San Felipe Hidalgo, where corn crops are intermingled within forested areas, and [3] an urban site in Nanacamilpa, a city with more than 10,000 inhabitants (Fig 1).

### Sampling protocols

We established two sampling sites on each of the three locations of the landscape gradient and captured bats in the rainy and dry seasons. In each sampling site, we set two 12 m mist nets for three consecutive nights, from 18:00 h to midnight, and checked for netting bats each half hour. Once a bat was trapped, we passed a sterile cotton swab along its back and wings [19]. Then, we released bats from the net, and species, sex, body measurements, and weight were annotated for all captured individuals. Bat species were identified using a field guide for bats [26]. The capture and handling of individuals followed guidelines for the use of wild mammals in research of the American Society of Mammalogy [27]. The collecting permit was issued by the General Administration of Wildlife of Mexico (SGPA/DGVS/04283/17 and SGPA/DGVS/09/K41731/10/19).

The collected cotton swabs were stacked in a dry cooler at 4–6°C and transported to the laboratory. Cotton swabs were washed with 0.5% sterile tween 80. Each swab was vortexed for one minute three times to allow the fungal propagule to separate due that conidia could be hydrophobic or hydrophilic. This procedure facilitates obtaining the highest number of fungal propagules. Then, 200 μL of this conidial suspension was deposited by extension with a glass rod in Petri dishes. Rose Bengal Agar with chloramphenicol (500 mg/L), and Czapek Agar (Merck, Mexico) were used as substrates. Both are isolation media that are fungal specific. To isolate psychrotrophic and mesophilic fungi, we kept two samples for 7 to 11 days at 4°C, and 28°C, respectively. After incubation time, the macro and micromorphology of the fungal colonies obtained in each media were described. Each colony was considered to come from a propagule: conidium, yeast, hyphal fragment or any structure that could give rise to growth and identified as a Colony Forming Unit (CFU). For macromorphological identification, color, morphology and production of pigments and exudates were described [28]. Glass slide preparations stained with cotton blue and observed with an Olympus 5640 phase-contrast microscope. Identification was based on the Huges Tubaki Barron conidial ontogeny taxonomic system. The observed structures were compared with dichotomous keys [29,30]. The chemicals used in the laboratory procedures were obtained from Sigma-Aldrich Química (Toluca, Mexico).

### Data analyses

We used a multivariate abundance analysis for bat abundances. This approach allows fitting models based on methods of general linear models (GLM) [31]. To perform the analysis, we assumed a negative binomial distribution to fit the model, as our dataset contained many zeros, and used sites as random effect to determine the effect of the treatments; statistical significance was calculated with a likelihood-ratio Wilks test. Given that our data did not show homogeneity of variance, we used deviance as a dispersion metric. We also included a GLM model to test differences in both bat and fungal species diversity, using the same assumptions as with the multivariate abundance analysis. A non-metric multidimensional scaling (NMDS) was produced to order bat and fungal communities according to their structure and performed an ANOSIM to evaluate differences in species grouping. We used the Shannon and Simpson (1-D) diversity indexes (as both complemented each other), and individual-based accumulation curves to estimate changes in bat and fungal diversities of sampling sites along the landscape gradient. Diversity analyses were constructed based on Hill numbers, given its effectiveness for temporal and spatial biodiversity estimation and comparison of the species diversity from different assemblages [32]. In addition, we conducted a principal component analysis to order bat species according to their fungal diversity, with RStudio (v.4.02) using the MVAbund [31], and iNext [33] libraries.

## Results

**Fig 2:**
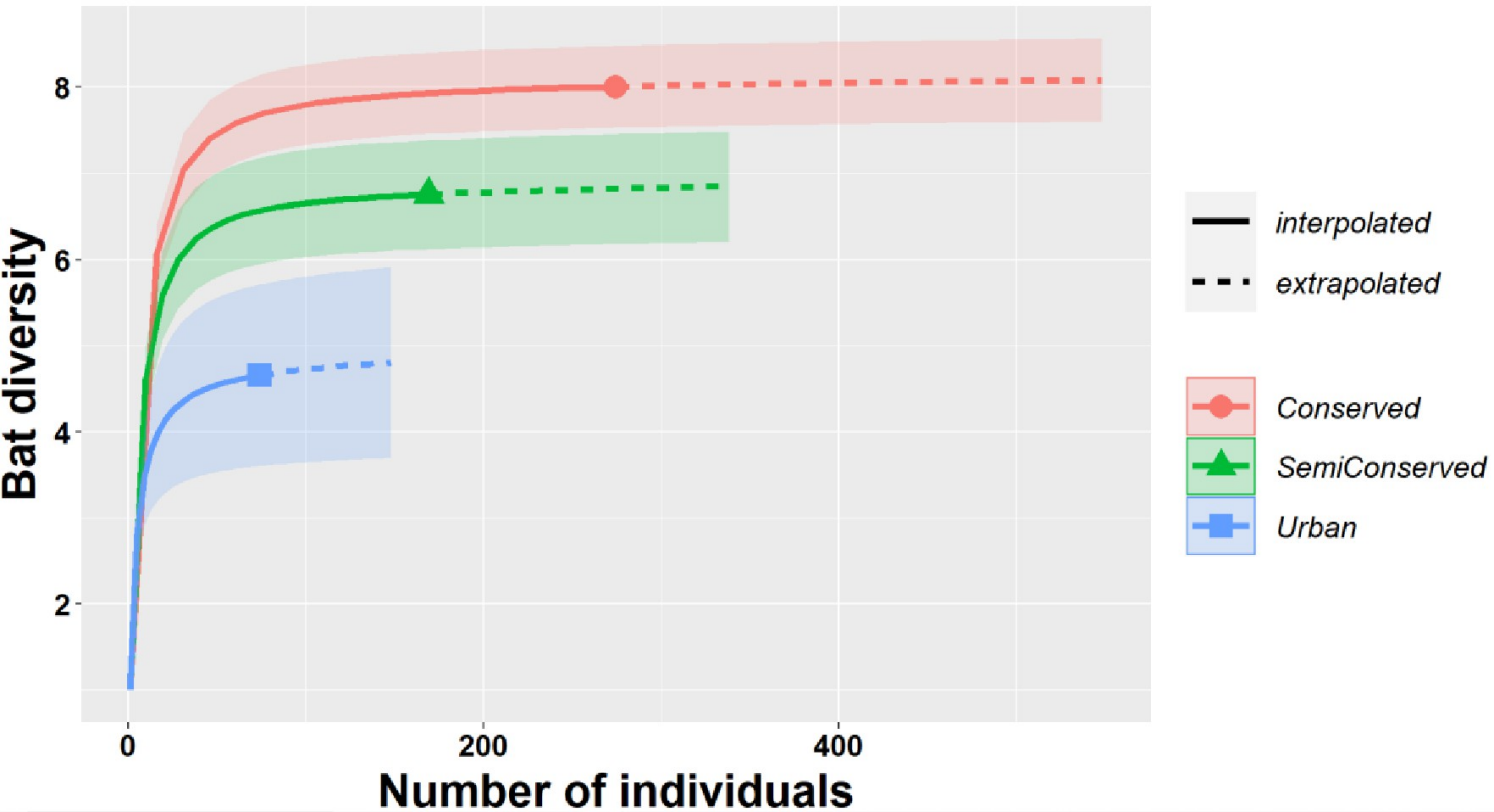
Bat species accumulation curves showing the rarefaction and predicted alfa species diversity along the landscape gradient.

A total of ten bat species were captured, where *Eptesicus fuscus* and *Corynorhinus townsendii* were the most and least abundant, respectively (Table 1). We observed significant differences in bat abundances among localities (Wilks = 165.3, *p = 0*.*0001;* Deviance = 15.3). Bat species showing significant higher abundances in conserved sites were *Myotis ciliolabrum* (Wilks = 42.08, *p = 0*.*001*; Deviance = 52.488), and *C*. *townsendii* (Wilks = 22.76, *p = 0*.*001*; Deviance = 38.488); the latter two species were not recorded in urban sites. Bat species showing significant higher abundances in urban sites were *E*. *fuscus* (Wilks = 183.82, *p = 0*.*004*; Deviance = 18.859), *Myotis velifer* (Wilks = 175.70, *p = 0*.*012*; Deviance = 13.996), and *Lasiurus cinereus* (Wilks = 132.68, *p* = 0.001; Deviance = 34.72). We observed significant differences in Shannon (Z = 10.23, *p = 0*.*001*) and Simpson (Z = 7.73, *p = 0*.*001*) bat diversity between conserved and urban sites, which is represented by individual-based species accumulation curves (Fig 2).

**Table 1:** List of fungi genera associated with species of bats recorded in the three study sites along a landscape gradient of conserved, semi-conserved and urban habitats in the Nanacamilpa municipality, in the State of Tlaxcala in central Mexico.

Fungi genera	*Corynorhinus townsendii*	*Eptesicus fuscus*	*Lasiurus cinereus*	*Lasiurus intermedius*	*Myotis auriculus*	*Myotis californicus*	*Myotis ciliolabrum*	*Myotis velifer*	*Natalus lanatus*	*Parastrellus hesperus*
*Acremonium*		X	X	X	X	X	X		X	X
*Alternaria*		X	X	X	X	X	X	X	X	X
*Aspergillus*	X	X	X	X	X	X	X	X	X	X
***Aureobasidium***						**X**				
*Beauveria*		X	X	X	X	X	X	X	X	X
***Bispora***			**X**	**X**				**X**		**X**
*Cladosporium*	X	X	X			X	X	X	X	X
*Fusarium*			X	X		X		X		
*Penicillium*	X	X	X	X	X	X	X	X	X	X
*Biospora 2*		X	X		X	X	X		X	X
*Scopulariopsis*	X	X		X	X	X	X	X	X	X
***Stachybotrys***					**X**			**X**		
*Trichoderma*		X	X	X	X	X	X	X	X	
***Verticillium***					**X**					

**Fig 3:**
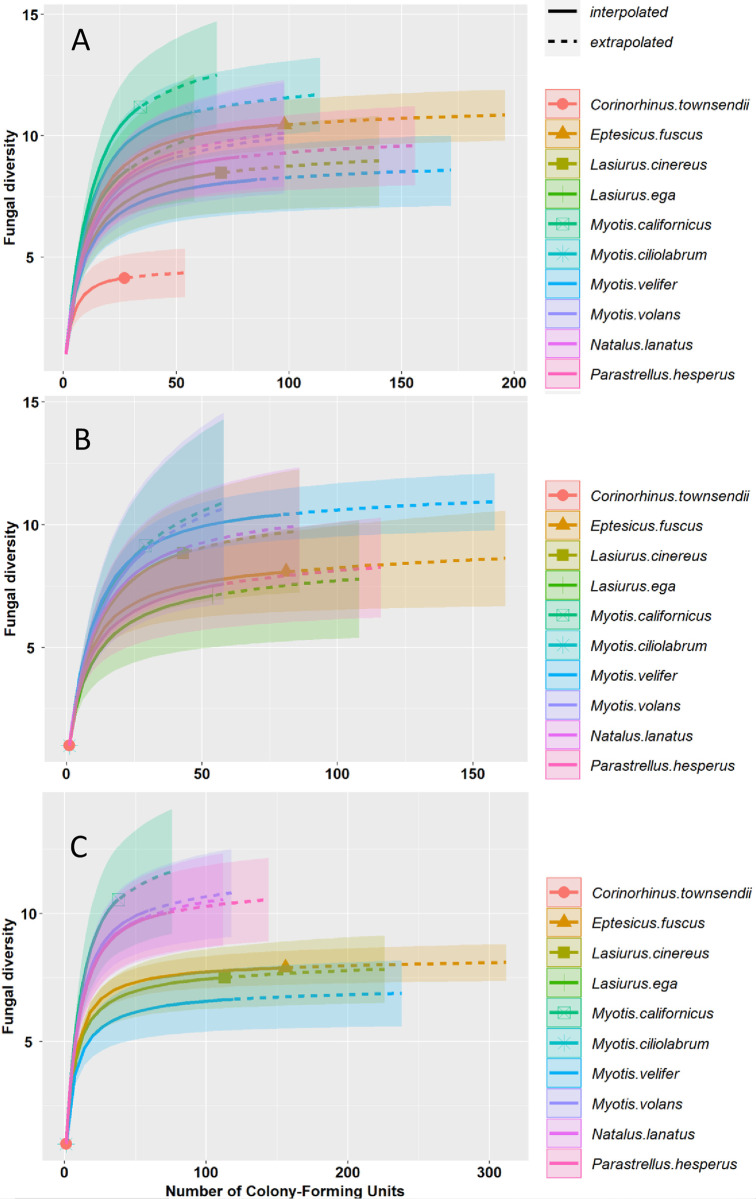
Fungi genera accumulation curves associated with bat species showing the rarefaction and predicted species diversity in conserved site (A), semi-conserved site (B), and urban site (C).

A total of 15 culturable fungi genera included the fungal diversity associated to bats; nine fungi genera were shared along the landscape gradient (Table 1). *Penicillium* (2314 CFU) and *Aspergillus* (329 CFU) were the most abundant genera, and *Aureobasidium*, *Bispora*, *Stachybotrys* and *Verticillium* were only documented in the conserved sites (12 CFU among all). Overall, we observed significant differences in fungal abundances in bats along the landscape gradient (Wilks = 77.3 *p = 0*.*0001*; Deviance = 112.3). *E*. *fuscus* (Wilks = 23.4, *p = 0*.*016*; Deviance = 13.85), *M*. *velifer* (Wilks = 192.2, *p = 0*.*012*; Deviance = 12.93), and *L*. *cinereus* (Wilks = 98.4, *p = 0*.*045*; Deviance = 10.12) showed a higher fungal diversity in urban sites. Further, we observed significant differences between the Shannon (Z = 13.16, *p = 0*.*01*) and Simpson (Z = 21.75, *p = 0*.*001*) diversity of fungal diversity in bats along the landscape gradient. The individual site-based accumulation curves showed changes in the fungal diversity associated with the sampling sites. *M*. *californicus* and *M*. *velifer* showed the highest fungal diversity in conserved and urban sites, respectively (Fig 3).

**Fig 4:**
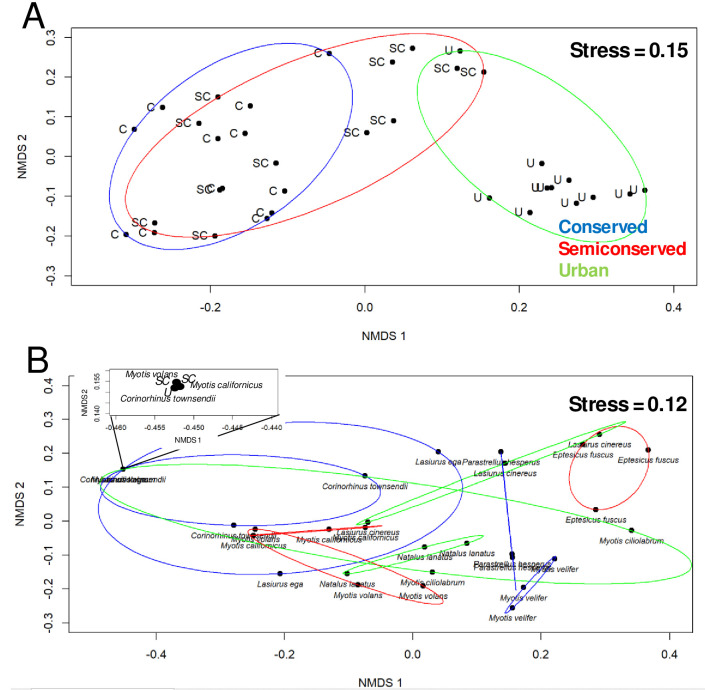
Multidimensional scaling analyses depicting sites ordination according to their bat species composition (A) and ordination of bat species related with fungal species (B) along the landscape gradient. C = conserved, SC = semi-conserved, U = urban sites.

**Fig 5:**
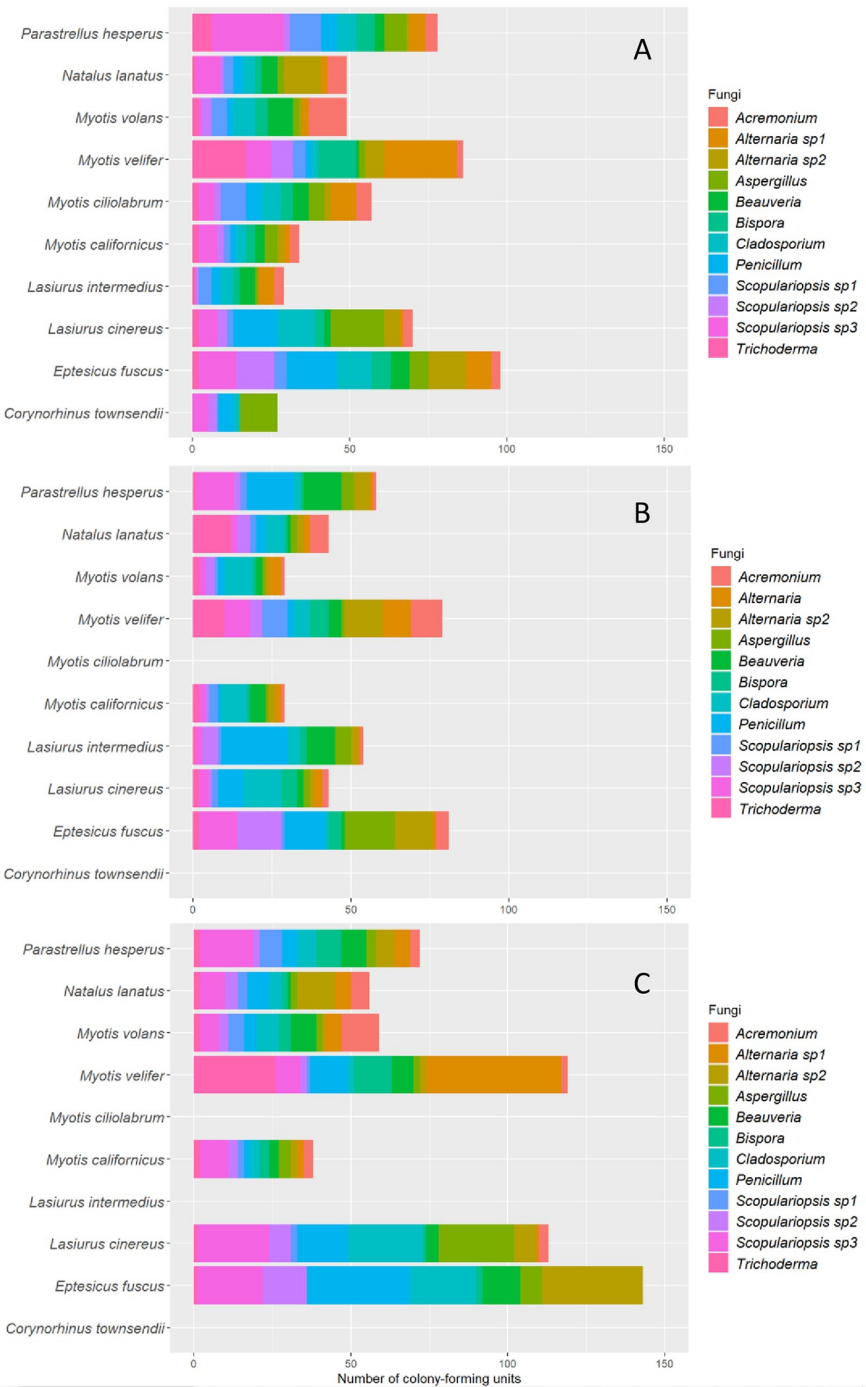
Relative abundances of genera in fungal diversity for each species of bat recorded during this study in conserved (A), semi-conserved (B), and urban (C) sites, respectively.

Changes in both bat and fungi communities were reflected in the NMDS analysis. Species bat composition was influenced in the ordering of sites. (Fig 4A). Conserved (C) and semi-conserved (SC) sites were significantly different from urban sites (ANOSIM statistic R = 0.58, *P* = 0.001). On the other hand, fungal community composition determined the ordination of bat species, although the species grouping was more complex. Bat species constituted several groups of which most were composed of the same species but with changes in a multidimensional space, according to its fungal load (Fig 4B). These groups showed a significant difference (ANOSIM statistic R = 0.58, *P* = 0.001). This change was closely associated with fungal abundance variations by the sampling sites in the landscape gradient (Fig 5).

## Discussion

We expected a decrease in both bat and fungal diversities associated with the conserved, semi-conserved, and urban landscape gradient. Despite that our results coincided with this expectation, changes in bat and fungal species richness had different origins. For example, we documented a total of 10 bat species in conserved sites, and a reduction to eight, and seven bat species in semi-conserved and urban sites, respectively, due to the absence of *L*. *intermedius*, *M*. *melanorhinus*, and *C*. *townsendii*. A similar bat species richness was observed at La Malinche protected area, adjacent to our conserved sites [34]. These authors attributed that bat species composition at their sites was determined by climatic variables and water availability [34]. In this regard, the low proportion of insectivorous bat species that we observed in urban sites can be attributed to the fact that these bats are sensitive to food and roost availability, which appear to be restricted in this habitat [23]. On the other hand, *C*. *townsendii* is a solitary rare species sensitive to human-induced habitats [35,36]. The diet of this bat is based on nocturnal butterflies and moths, which become less abundant in urban and semi-urban areas [37]. Thus, we suspect that the absence of the latter three species of bats in our urban and semi-conserved sites could be explained by a low insect availability and climatic variability typical of urban sites [38].

We also expected associations along our landscape gradient, with a decreasing fungal diversity occurring in conserved, semi-conserved, and urban sites, respectively. We documented several genera of fungi shared between bat species showing different abundances along the landscape gradient. This nonrandom pattern appeared to be related to the spatial associations between fungi and bat [host] species, as documented in hummingbirds [39]. For example, the fungal species richness in *E*. *fuscus* included nine genera in conserved sites to four dominant genera in urban sites, represented by *Trichoderma*, *Bispora*, *Penicillium* and *Alternaria* sp2. These four genera have been reported as airborne fungi in urban environments in Mexico; roosting bats in urban area are thus propense to harbor these fungi genera in their skins [40,41].

In contrast, *M*. *velifer* only showed two abundant fungi genera, represented by *Trichoderma* and *Alternaria* sp2, which can be harbored when bats roost and feed on plants. These two fungi genera are generally found on plants, although *Trichoderma* sp, is an endophyte also known as a bio-fungicide, as well as promoting plant growth [42]. It is known that *Tricoderma* contains enzymatic and chemical defense mechanisms as mycotoxins [43], and more than 100 metabolites with antibiotic activity including polyketides, pyrones, terpenes, and metabolites derived from amino acids, and polypeptides. *Alternaria* sp has a widespread distribution, and is reported to contain a variety of biological activities such as phytotoxic, cytotoxic, and antimicrobial properties [44]. Further, some *Alternaria* strains can use keratin as a carbon source, which is found in the hair of bats, and can serve as a source of nutrients for the fungus [45]. *Penicillium* is a widespread fungus and one of the most diverse fungi groups and has been associated with plants on vegetative tissues and endophytically as well.

Other studies also have documented similar trends of fungal diversity associated with bats. For instance, in the Amazon basin were documented nineteen fungi species in several tissues of 71 Neotropical bat species [46]. In Pernanbuco, Brazil, were documented forty-five species of fungi on bat wings of the Neotropical bats *Carollia perspicillata* and *Diphylla ecaudata* [19]. In Neartic bats, as *Myotis sodalist*, *Perimyotis subflavus*, and *Myotis septentrionalis*, several genera of fungi as *Cladosporium*, *Fusarium*, *Geomyces*, *Mortierella*, *Penicillium*, and *Trichosporon* were commonly found on wings of bat [47].

Our study documented that fungal diversity in bats is composed by species reported as free-living in the environment or associated with plants [46]. Most fungal genera documented are commonly distributed in temperate forests. Thus, documented changes of fungal loads in bats are remarkable, given that fungal diversity was not only limited by environment conditions [8,11–10] but also by activity of species of bats. In addition to previous studies that have documented associations of species of fungi and bats [10,13,18,48,49], our study contributed to quantifying these species interactions along a landscape gradient including conserved, semi-conserved and urban habitats. Further, biogeographic patterns of fungal communities are related to land use, soil management, vegetation, and pollutant concentrations [52]. Even though the fungal genera reported in this study can be considered cosmopolite, our findings contribute to the understanding of the preference of the fungal groups to different habitats, related to a decreasing conservation landscape gradient. For example, phytopathogenic fungi such as *Alternaria* or *Cladosporium*, will increase as habitat disturbance increases, especially with changes in land use [50], mainly when they are destined for agricultural or urban activities.

Fungi species associated with faunistic groups are typically considered zoonotic or environmental pathogens [51,52] or for transporting spores [53], although little is known on their ecological role in their hosts (but see 39). We reported changes in fungal diversity related to changes in bat diversity associated with a landscape gradient. Further research needs to incorporate monitoring of fungi and bat diversity in more ecosystems for a better understanding of the relationship between fungal and bat diversities and species interactions.

## Supporting information

S1 File (PDF)
