# Supplementary material for: Fungal and bat diversities along a landscape gradient in central Mexico
Source: PLoS One. 2024 Sep 9;19(9):e0310235. doi: 10.1371/journal.pone.0310235 (PMC11383230; doi:10.1371/journal.pone.0310235)
Supplement: S1 File — (PDF) [file pone.0310235.s001.pdf]

Supplementary material

Bat and fungal data documented in the whole study area

Landscape	Fungi genera	*Corynorhinus townsendii*	*Eptesicus fuscus*	*Lasiurus cinereus*	*Lasiurus intermedius*	*Myotis californicus*	*Myotis ciliolabrum*	*Myotis velifer*	*Myotis volans*	*Natalus lanatus*	*Parastrellus hesperus*
Urban	*Acremonium*	0	1	3	0	3	0	2	12	6	3
Urban	*Alternaria sp1*	0	12	1	0	2	0	43	5	5	5
Urban	*Alternaria sp2*	0	32	7	0	2	0	2	1	12	6
Urban	*Aspergillus*	0	7	24	0	4	0	2	2	2	3
Urban	*Beauveria*	0	12	4	0	3	0	7	8	1	8
Urban	*Bispora*	0	2	1	0	3	0	12	4	2	8
Urban	*Cladosporium*	0	21	24	0	3	0	2	7	4	6
Urban	*Penicillum*	0	33	16	0	2	0	12	4	7	5
Urban	*Scopulariopsis sp1*	0	0	2	0	2	0	1	5	3	7
Urban	*Scopulariopsis sp2*	0	14	7	0	3	0	2	3	4	2
Urban	*Scopulariopsis sp3*	0	21	23	0	9	0	8	6	8	17
Urban	*Trichoderma*	0	1	1	0	2	0	26	2	2	2
Semi_Conserved	*Acremonium*	0	4	2	1	1	0	10	1	6	1
Semi_Conserved	*Alternaria*	0	1	3	1	2	0	9	4	2	1
Semi_Conserved	*Alternaria sp2*	0	12	1	2	2	0	12	1	2	5
Semi_Conserved	*Aspergillus*	0	16	2	5	1	0	1	1	2	4
Semi_Conserved	*Beauveria*	0	1	2	9	5	0	4	2	1	12
Semi_Conserved	*Bispora*	0	4	5	2	1	0	6	1	1	1
Semi_Conserved	*Cladosporium*	0	1	12	4	8	0	5	9	6	2
Semi_Conserved	*Penicillum*	0	13	8	21	1	0	2	2	3	15

Semi_Conserved	*Scopulariopsis sp1*	0	1	2	1	3	0	8	1	2	2
Semi_Conserved	*Scopulariopsis sp2*	0	14	1	5	1	0	4	3	4	2
Semi_Conserved	*Scopulariopsis sp3*	0	12	3	2	2	0	8	2	2	12
Semi_Conserved	*Trichoderma*	0	2	2	1	2	0	10	2	12	1
Conserved	*Acremonium*	0	3	3	3	3	5	2	12	6	4
Conserved	*Alternaria sp1*	0	8	1	5	2	8	23	2	2	5
Conserved	*Alternaria sp2*	0	12	5	0	2	2	6	1	12	1
Conserved	*Aspergillus*	12	6	17	1	4	5	2	2	2	7
Conserved	*Beauveria*	0	6	2	5	3	5	1	8	5	3
Conserved	*Bispora*	0	6	3	2	3	4	12	4	2	6
Conserved	*Cladosporium*	2	11	12	4	3	6	2	7	4	6
Conserved	*Penicillum*	5	16	14	3	2	5	2	2	3	5
Conserved	*Scopulariopsis sp1*	0	4	2	4	2	8	4	5	3	10
Conserved	*Scopulariopsis sp2*	3	12	3	1	2	2	7	3	1	2
Conserved	*Scopulariopsis sp3*	5	12	6	0	6	5	8	1	8	23
Conserved	*Trichoderma*	0	2	2	1	2	2	17	2	1	6
